# Intradural extramedullary primary hydatid cyst of the spine: a case report and review of literature

**DOI:** 10.1007/s00586-012-2373-1

**Published:** 2012-06-16

**Authors:** Iraj Lotfinia, Sima Sayyahmelli, Ata Mahdkhah, M. M. Shoja

**Affiliations:** 1 Neuroscience Research Centre, Shohda Hospital, Tabriz University of Medical Sciences, Tabriz, Iran; 2 Shohda Hospital, Tabriz University of Medical Sciences, Tabriz, Iran; 3 Tuberculosis and Lung Diseases Research Center, Tabriz University of Medical Sciences, Tabriz, Iran

**Keywords:** Hydatid cyst, Spine, MRI, Surgery, *Echinococcus granulosus*

## Abstract

Primary intradural extramedullary hydatid cyst is a rare form of parasitic infection, causing focal neurological signs, commonly observed in sheep-raising areas of the world. We report a rare case of intradural, extramedullary spinal cyst, which we had misdiagnosis in the first surgery, because of rarity of the case. A 55-year-old man presented to our hospital in August 2008. He was admitted to our clinic because of lumbar pain of increasing severity and progressive difficulty with walking and stiffness of both lower limbs, which had lasted for 1 month. On the basis of imaging results, arachnoid cyst of the lumbar spine was diagnosed. Due to rapid progression of the patient’s symptoms toward spastic paraplegia, he underwent an emergency surgical decompression procedure. The patient underwent exploratory surgery using a posterior approach. A L1–L2 laminectomy was performed. After opening the dura, an intradural extramedullary cystic mass was determined. The surgical specimen measured 6 × 2 cm and was described as a whitish, pearl-like, semitranslucent, cystic material, which was thought to be parasitic. Surgery has to be followed by albendazole therapy.

## Introduction

Hydatid is a Greek word meaning “watery cyst”. Hydatid disease is caused by the parasitic tapeworm Echinococcus (*E. granulosus*, and less commonly *E. multilocularis*) [1]. Hydatidosis caused by *Echinococcus granulosus* is an endemic parasitic disease in Mediterranean areas, such as North Africa, Spain, Greece, Turkey, Portugal, Middle East, Australia, New Zealand, South America, Baltic areas, the Philippines, Northern China and the Indian sub-continent [2]. Although the disease is rare in Western Europe and North America [3], but has a worldwide distribution [4] and given the ease of modern travel Echinococcus is a worldwide problem [5].

The most common sites of infection are liver (75 %), lung (15 %), brain (2–4 %), and genitourinary tract (2–3 %) [1]. Only 0.5–3.1 % of patients suffer from bone involvement, half of which occurs in the spine [3]. Spinal hydatid cysts are located most commonly at the thoracic (52 %), followed by the lumbar (37 %) and then the cervical and sacral levels [1, 6]. Spine is the most common site for the hydatid disease of the bone [1]. Spinal hydatid cysts account for 1 % of all cases of hydatid disease [7]. Braithwaite and Lees [8] have classified the spinal involvement by hydatid disease in five groups: (1) primary intramedullary hydatid cyst; (2) intradural extramedullary hydatid cyst; (3) extradural intraspinal hydatid cyst; (4) hydatid disease of the vertebrae; (5) paravertebral hydatid disease. The first three types in this group are considered rare. Intradural presentation is extremely rare [6, 7, 9]. Although secondary intradural involvement can occur as a consequence of spinal dural injury or also via spread through the subarachnoid space of a ruptured intracranial cyst, primary intradural extramedullary hydatid cysts are extremely uncommon [1, 6, 10, 11]. In this paper, we report one case of primary intradural extramedullary hydatid cyst and review literature about the reported cases.

## Case report

### Presentation and examination

A 55-year-old man presented to our hospital in August 2008. He was admitted to our clinic because of lumbar pain of increasing severity and progressive difficulty with walking and stiffness of both lower limbs, which had lasted for 1 month.

At that time, he had been complaining of weakness and numbness in both lower limbs, and during the 4 days prior to his presentation at the hospital, he had experienced difficulty with walking. Neurological examination had revealed bilateral lower-extremity weakness and spastic paraparesis. No sphincter disturbances were demonstrated.

He noted neither weight loss nor fever during the last month. A general physical examination revealed no abnormality. His erythrocyte sedimentation rate and C-reactive protein levels as well as the results of other biochemical analyses were normal. The results of all hematological tests had been normal. Chest X-ray study revealed normal findings.

### Neuroimaging

**Fig. 1:**
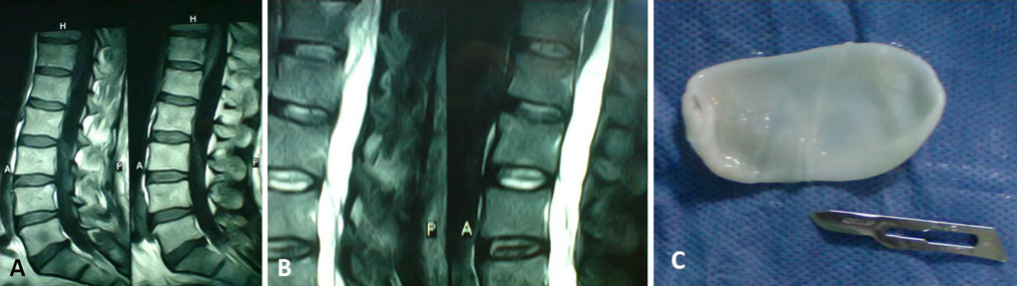
**a**, **b** Magnetic resonance imaging of the lumbar spine showed a cystic lesion compressing the thecal sac, involving the L1–L2. **c** A classic milky-white cyst wall was excised

Magnetic resonance imaging of the lumbar spine showed a cystic lesion compressing the thecal sac, involving the L1–L2 (Fig. 1a, b). The lesion was hypointense on T1-weighted images and hyperintense on T2-weighted images. The body of the L1 and L2 vertebrae appeared to be intact. The paraspinal muscles were shown to be intact.

### Operation and histopathological examination

On the basis of imaging results, arachnoid cyst of the lumbar spine was diagnosed.

Due to rapid progression of the patient’s symptoms toward spastic paraplegia, he underwent an emergency surgical decompression procedure. The patient underwent exploratory surgery using a posterior approach. A L1–L2 laminectomy was performed. After opening the dura, an intradural extramedullary cystic mass was determined. The surgical specimen measured 6 × 2 cm and was described as a whitish, pearllike, semitranslucent, cystic material, which was thought to be parasitic.

The cyst was initially aspirated and clear fluid was found. This clear fluid strongly indicated a possibility of hydatid disease; therefore, 3 % hypertonic saline was used to irrigate the operative site and was injected into the cysts. A classic milky-white cyst wall was encountered, confirming our suspicion of a hydatid cyst. The cyst was excised during concurrent steroid therapy to avoid an anaphylactic reaction (Fig. 1c). The entire operative field and surrounding regions were irrigated with hypertonic saline. The wound was closed in layers.

In the histopathological examination, the cyst wall was noted to stain with Hematoxylin and Eosin (H&E).

There were eosinophilic inflammatory cells in the cyst wall, which supported the presence of a heavy inflammatory reaction, as well as eosinophilic laminar membranous material located in granular eosinophilic debris, which supported a diagnosis of hydatidosis.

### Postoperative course

After the operation, the patient’s neurological status improved, and he was discharged with instructions to continue a regimen of anthelmintic treatment consisting of albendazole (400 mg twice a day).

The biopsy specimen results confirmed the diagnosis of hydatid cysts. Neither physical examination nor ultrasonographic examination of the abdominal and pelvic organs demonstrated any localization of disease other than in the spine. Postoperative recovery was uneventful. Over the next 5 months, he made a complete neurological recovery.

### Repeated presentation and examination

The patient returned 6 months later; his chief symptom was back pain that had continued for 1 month, associated with numbness in his bilateral lower extremities. Clinical findings included 4/5 motor strength in the both lower extremity causing a spastic gait. No sphincter disturbances were demonstrated.

### Findings on repeated neuroimaging

**Fig. 2:**
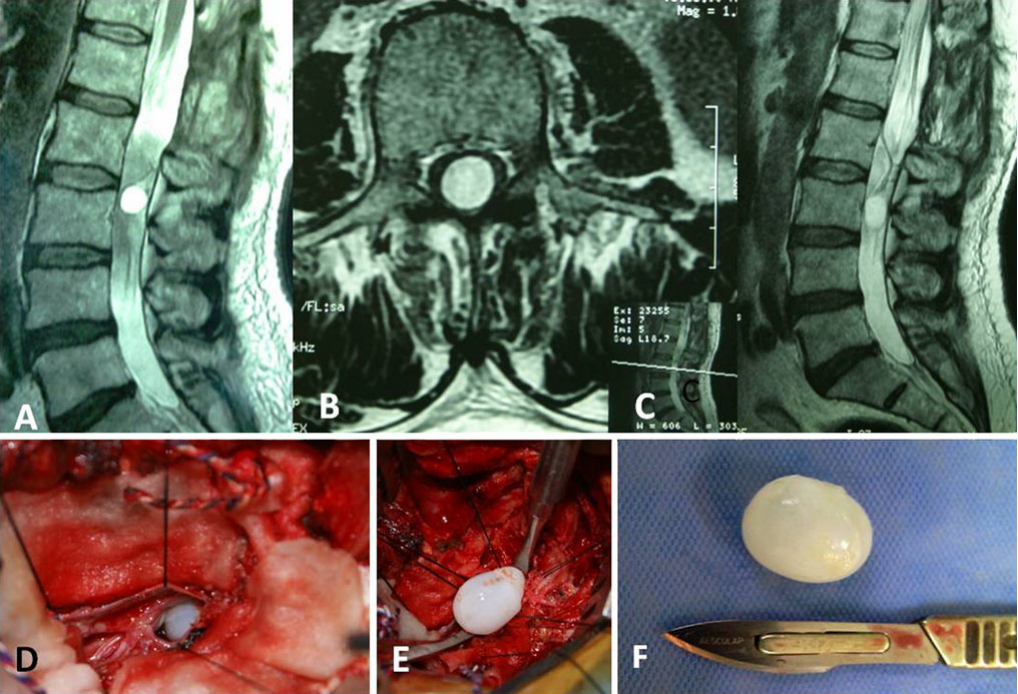
**a**–**c** On repeated MR imaging, a cystic structure was demonstrated at the L3 level, a level below the prior operated level. **d**–**f** After opening the dura, an intradural extramedullary cystic mass was determined. The lesion was removed gross totally

On repeated MR imaging, a cystic structure was demonstrated at the L3 level, a level below the prior operated level (Fig. 2a–c). The lesion was hypointense on T1-weighted images and hyperintense on T2-weighted images. The postoperative changes were demonstrated at the L1–L2 level, due to prior laminectomy. The diagnosis of hydatid cyst was made on the basis of MRl findings and the previous history.

### Second operation and postoperative course

A L3 laminectomy was performed. After opening the dura, an intradural extramedullary cystic mass was determined. The lesion was removed gross totally and the cavity was irrigated with hypertonic saline (Fig. 2d–f).

Histopathological examination revealed an amorphous acellular cuticular lesion compatible with hydatid cyst.

Albendazole treatment was started in the early postoperative stage and three cycles of treatment (each cycle consisted of 15 mg/kg/day albendazole for 4 weeks) were completed. The postoperative stage was unremarkable without any additional problem.

The patient is currently in his third year of treatment, he has experienced no additional problems. All biochemical values have remained within normal limits during follow-up.

## Discussion

Primary intradural extramedullary hydatid cyst is a rare form of parasitic infection, causing focal neurological signs, commonly observed in sheep-raising areas of the world [12]. Cases of cystic hydatid disease are mostly found in areas where dogs and livestock coexist. Although infrequent, infections by cestodes constitute a cause of disease in HIV-infected patients, especially in endemic areas [13]. Adult worms mature in the intestine of the dog, wolf, and other carnivorous animals (definitive host), and the eggs are released in the feces. Intermediate hosts such as sheep and cattle ingest the eggs. Sometimes, humans act as an intermediate host, and contract the disease by means of contamination through the direct contact with the definitive host or its feces or by ingesting food infected with parasite eggs [14]. Once within human or any other intermediate host, the ingested eggs hatch in the duodenum to release oncospheres (true larvae) that burrow into the jejuna submucosa and enter veins or lymphatic vessels [2]. They reach the liver, which acts as an effective filter for most of the larvae. However, if that barrier is overwhelmed, the larvae pass through the inferior vena cava into the right side of the heart and then to the lungs. If the worm is not lodged in liver or lungs, it may be trapped virtually anywhere in the body, such as peritoneum, spleen, kidney, heart, brain, spine, bony skeleton and muscles [3]. The second reported method of human infestation is by inhaling dust carrying dried ova, which hatch in the lungs and form a cyst there [15]. The metacestode cyst develops over a course of years in the patient [16]. Not every larva develops; over 90 % are eliminated by the host reaction. The cysts consist of an outer fibrous layer and a cestode-derived inner germinal membrane containing scolices [6, 17]. Human beings are accidental intermediate hosts. Spinal hydatid cysts account for 1 % of all cases of hydatid disease [7]. Hydatid cysts are usually located at the thoracic level (52 %), followed by the lumbar (37 %), and then the cervical and sacral levels [7]. It affects both sexes equally, but is seen more often in the younger population [7]. The route of spread of the disease to the spine is still not fully determined. Schroeder and Medoc [18] believes that the hexacanth embryo reaches bone along a tortuous and complicated systemic circulatory route passing through the liver and lungs. Based on the view of Hernigou [19], spine involvement is more than other bones. This peculiarity is a result of the presence of the Batson venous plexus, in which the blood flows directly from the intestinal vascular bed to the spine without being filtered through the liver or lungs. Paradoxic emboli through that system may be the reason for the occurrence of spine hydatidosis. The other way of spine involvement may be direct extension of a hepatic or pulmonary focus [20]. Intradural extramedullary hydatid cyst can be classified into two groups; primary, in which there is no other site for spreading of parasite to central nervous system. In this form, primary hematic dissemination is regarded as the most plausible mechanism [21]. Investigators believe that approximately 15 % of them pass these barriers and lodge somewhere else in the body [15], and these oncospheres may reach any part of the body such as intradural space. Ley and Marti [22], to explain the intradural location of the cysts secondary to pulmonary location as seen in their patient, postulated that the parasite embryo circulating in the bloodstream enters through an intercostal artery. The secondary form is usually due to rupture of intracranial hydatid cyst and spreading via subarachnoid space during surgery of spontaneously, or spreading from adjacent structures such as spine involvement. Dural tearing during surgery or during an aggressive invasion of spinal space, like lumbar puncture, may have a role in the etiology of the spreading of the infestation into the intradural space [21]. Within 3 weeks, the embryo develops into a larva, and a cyst is formed [23].

Histopathologically, three layers can be identified in the wall of the hydatid cyst: a peripheral adventitial layer which consists of fibrous tissue containing many eosinophils, an intermediate cuticular layer containing amorphousdensely staining laminated chitinous material, and an innergerminal layer that contains nucleated epithelium. It is the germinal layer that gives rise to brood capsules, and scolices (the larval stage of the parasite) develop within these vacuolated structures. The broad capsules (daughter cysts) eventually detach and float freely in the fluid of the hydatid cyst. The number of scolices increases within the daughter cysts over time, causing enlargement of the cyst. The pathology of hydatid disease is caused by the mass effect of the growing cyst [24] so symptoms of spinal hydatid disease are nonpathognomonic, and symptoms are usually related to compression of the spinal cord [7]. Patient may present with symptoms and signs related to spinal cord compression, and due to the relative rarity of the problem the diagnosis was frequently made during surgery [3]. In general, the first symptoms are backache and radicular pain, weaknesses of the limbs occurs in the later phase of the disease [6]. Radiological studies are usually inconclusive but may be helpful in the diagnosis of hydatid disease. Laboratory tests such as ELISA, indirect hemagglutination and complement fixation tests are reported to be 80–100 % sensitive and 88–96 % specific in abdominal disease [3]. However, the sensitivity decreases abruptly to 25–56 % in extrahepatic disease which limits their use in the diagnosis or follow up for primary bone disease [3]. The best preoperative diagnostic procedure is MR [23, 51].

Berk et al. [41] reviewed MRI characteristics of the lesions and concluded that they had a unique appearance: a sausage-like shape with two dome-shaped ends, thin and regular walls and no septation or debris in the lumen. The lesions are occasionally spherical. Signal characteristics of the cyst content are usually similar to that of CSF. On T1-weighted images, the cyst wall may be isointense or give slightly lower signal than its contents, and T2-weighted images demonstrates a low-intensity rim surrounding the homogeneous high-signal cyst content. The T2-weighted image features indicate the viability of the cyst: a decrease in high signal and increase in low signal from collapsed cyst walls indicate a succumbed cyst. The cyst wall demonstrates slight contrast enhancement. The low-signal rim on T2-weighted images results from reactive fibrosis and degeneration around the parasitic membrane and correlates with the histopathological examination [41]. MRI is also very helpful in early diagnosis of postoperative recurrence. The management of hydatid cyst is always surgery; treatment is mainly total removal without cyst rupture [1, 14]. Careful dissection and irrigation around the cyst is essential to avoid intraoperative cyst rupture, which leads to recurrence [52, 53]. In addition to recurrence, rupture of a cyst with spillage of the content may provoke a variety of hypersensitivity reactions such as pruritus, urticaria, edema, dyspnea, asthma, vomiting, diarrhea, colicky abdominal pain and even anaphylactic shock [3]. Posterior approach is usually preferred for pure intradural or pure epidural lesions at all the spinal levels [1]. If cysts rupture during intervention, the surgical area should be irrigated with hypertonic saline; however, this seems relatively ineffective, since if any cyst ruptures into the intradural space, recurrence is inevitable [40]. Reports also indicate that irrigating the wound with hypertonic saline or a diluted betadine solution after cyst removal helps osmotically destroy and disrupt the parasites, although this remains unproven [4]. Adjuvant antihelmintic chemotherapy is essential to control the disease locally, to avoid systemic spread, and to prevent recurrence [54, 55]. The poor prognosis may be related to the localization (intradurally or extradurally) of the cyst and weak penetration of antihelminting drug, albendozole, and metabolites into the intradural space by a passive diffusion transport mechanism [51]. Benzimidazoles (albendazole or mebendazole), given alone or combined with praziqantel, are currently used for the treatment of hydatidosis. It should be kept in mind that both of these drugs are teratogenic and embryotoxic, and both may cause alterations in liver function and hematological adverse reactions [3]. Albendazole is preferred over mebendazole due to better pharmacokinetic properties and superior efficacy against helminths [3]. Their action is likely to be parasitostatic rather than parasiticidal [14]. A course of albendazole (800 mg daily in two divided doses) is continued for 1 to 6 months (usually 3 months) [3].

Recurrence is very rare with the intradural extramedullary form of hydatid disease [9]. We have to know cysts-containing hydatid sand are fertile cysts. If no sand is present, the cyst is sterile and does not form secondary cysts in case of spillage. It takes 6–9 months for a cyst to become fertile [56]. This delay is important to keep in mind. Patients who experience an intraoperative cyst rupture should be operated on again within that period [15].

In the literature review we found 44 another cases of primary intradural extramedullary hydatid cyst (Table 1), of which in mentioned cases 59.4 % man and 40.6 % women. The youngest patient was 4 and the oldest was 62 years old, and the mean age of reported cases was 20.5 years. So disease is more common in man and young patients. The most common clinical manifestation was paraplegia and after that were paraparesis, incontinence and radicular pain in lower limbs. Duration of symptoms is reported in nine cases, which varied between 1 to 24 months and the mean 5.2 months. The most common site for primary intradural extramedullary hydatid cyst was thoracic (46.5 %), lumbar (30.2 %), cervical (9.3 %) and then thoracolumbar, cervicodorsolumbar and cervicodorsal. Cysts occur in two types, unilocular and multilocular. Unilocular cyst is more common (57.5 %). In most reported patients there is no recurrence and symptoms improved after surgery.

**Table 1:** Cases of intradural extramedullary hydatid cyst which reported

Authors [References]	Age/sex	Origin	Symptom	Level	M/U	Postoperative course	Recurrence
Bartel (1928) [25]		France		Cervical	U		
Rauzier (1928) [25]		France		Cervical	U		
Valle (1928) [25]		France		Cervical, dorsal, lumbar	M		
Deve [26]		France		Cervical	U		
Deve [26]		France		Cervical	U		
Deve [26]		France		Cervicodorsolumbar	M		
Diez [27]		Argentina					
Bertrand et al. [28]	25/F	Morocco	Paraplegia	T11–T12	M	Improvement	Yes/after 13 months
Boixados [29]	4/F	Spain	Paraplegia	T5	U	Improvement	No
Dickmann [30]		Argentina					
Carrea and Murphy [31]	9/F	Argentina	Paraplegia	T4–T5	U	Improvement	No
Baurand et al. [32]	35/F	Nord	Paraplegia	T6	U	Improvement	No
Karvounis et al. [33]	37/F	Greece	Sciatica	L5–S1	M	Improvement	No
Bettaieb et al. [34]	4/M	Tunisia	Paraplegia	Dorsal high	U	Improvement	No
Bettaieb et al. [34]	8/M	Tunisia	Paraplegia	Dorsal high	U	Improvement	No
Bettaieb et al. [34]	8/M	Tunisia	Paraplegia	Dorsal medium	U	Improvement	No
Bettaieb et al. [34]	4/M	Tunisia	Paraplegia	Dorsal	U	Improvement	
Bettaieb et al. [34]	8/M	Tunisia	Paraplegia	Dorsal	U	Improvement	
Bettaieb et al. [34]	4/M	Tunisia	Paraplegia	Dorsal	M	Improvement	
Sánchez et al. [35]	62/F	Spain	Paraplegia	L2	M	Improvement	No
Sharma et al. [36]	14/M	India	Paraplegia	T6–T10	M	Improvement	No
Pamir et al. [37]	34/F	Turkey	Paraplegia	L2		No change	
Medjek et al. [38]	21/F	Algeria	Paraplegia	T12–L1	U	Improvement	No
Akhan et al. [39]	6/M	Turkey	Paraplegia incontinence 2 month	T9–T11	U		
İşlekel et al. [40]	19/M	Turkey	Paraplegia	L2–L4	M	Improvement	Yes
Berk and Erdogan [41]	17/M	Turkey	Paraparesis, incontinence, 1 month	T7–T8	M	Improvement	No
Kabbaj-El Kouhen et al. [42]	6/M	Morocco	Paraplegia	L1–L3	M	Improvement	
Chat et al. [43]	13/F	Morocco	Paraplegia	T5–T11 L4–L5	M	Improvement	
Pushparaj et al. [44]	40/F	India	Paraplegia	T10–T11	U	Improvement	No
Chakir et al. [25]	18/M	Morocco	Paraplegia	L1–L2	U	Improvement	Yes
Onbas et al. [9]	48/M	Turkey	Paraparesis	C7–T1	M	Improvement	Yes
Hilmani et al. [45]	25/F	Morocco	Cauda equina syndrome	L3–L5	M	Improvement	No
Kahilogullari et al. [6]	32/F	Turkey	Paraparesis, 6 months	L5–S2	M	Improvement	No
Erdoğmus et al. [46]	43/F	Turkey	Sciatica, 2 years	T12–L5	M		
Kalkan et al. [16]	8/M	Turkey	Paraparesis	T7–T8	U	Improvement	No
Shukla et al. [47]	8/M	India	Paraparesis	T12–L1	U	Improvement	No
Secer et al. [48]	34/M	Turkey	Paraparesis, 2 month	T11–T12	U	Improvement	No
Midyat et al. [49]	13/F	Turkey	Paraparesis	T10–T11	M	Improvement	No
Süslü et al. [50]	34/M	Turkey	Paraparesis				
Lakhdar et al. [7]	22//M	Morocco	Paraparesis, incontinence, 6 months	T11	M	Improvement	No
Lakhdar et al. [7]	5//M	Morocco	Cauda equina syndrome paresis,	T12–L1	M	Improvement	No
Lakhdar et al. [7]	10/F	Morocco	Paraparesis, incontinence, 1 month	L2–L5	U	Improvement	No
Arif and Zaheer [1]	9/M	Turkey	Paraparesis 4 months	T12–L3	U	Improvement	No
Shukla et al. [11]	5/M	India	Paraparesis, incontinence, 4 month	L4–S1	U	Improvement	No
Present case	55/M	Iran	Paraparesis, 1 month	L1–L3	U	Improvement	Yes

*M* vesicles multiple,* U* univesicle

## Conclusion

Vertebral hydatidosis is a rare but a serious condition, which is frequently misdiagnosed as arachnoid cyst, so in differential diagnosis of intradural arachnoid cyst especially in endemic area, hydatid cyst should be consider. A high index of suspicion combined with good-quality neuroimaging is important for early and correct diagnosis. Total surgical removal of the cysts without rupture should be aimed for. The best treatment remains active nationwide prevention of the disease.
